# Pediatric Langerhans Cell Histiocytosis: Unifocal Localization in the Frontal Bone Presenting With Periorbital Swelling

**DOI:** 10.1155/crpe/8892923

**Published:** 2025-05-24

**Authors:** Armela Gorica, Chiara Zeroli, Giulia Franzini, Diana Krepysheva, Giacomo Fiorita, Giuseppe Robustelli, Mario Turri Zanoni, Stefano Chiaravalli, Maddalena Marinoni, Paolo Castelnuovo, Maurizio Bignami, Francesca De Bernardi

**Affiliations:** ^1^ENT Department, ASST Sette Laghi, Insubria University, Varese, Italy; ^2^Pediatric Department, Filippo Del Ponte Hospital, Varese, Italy; ^3^Pediatric Oncology Department, IRCCS Foundation, National Institute of Tumor, Milan, Italy

## Abstract

**Background:** Langerhans cell histiocytosis is a rare disease, the pathogenesis of which remains a subject of debate, with considerations of either a neoplastic origin or an inflammatory nature. It arises from the uncontrolled proliferation of immature myeloid dendritic cells, leading to their accumulation in various sites. Recent studies indicate that Langerhans cell histiocytosis is a clonal neoplastic disorder driven by mutations in the MAP kinase pathway. When the bones are involved, the condition is often asymptomatic, but it can cause pain or soft tissue swelling, with the skull being the most affected site.

**Case Presentation:** We present the case of a 9-year-old boy who presented with painful left periorbital swelling. He initially underwent empirical medical therapy for suspected periorbital cellulitis. Due to a lack of improvement in symptoms, radiological assessment was subsequently performed, revealing frontoethmoidal-orbital bone resorption and a mass of ambiguous nature with peripheral enhancement in T1-weighted sequences. Following an ENT evaluation, a biopsy was performed using an endoscopic endonasal approach. The histological examination confirmed the presence of Langerhans cell histiocytosis, with unifocal localization in the frontal bone. Consequently, the patient began chemotherapy following the LCH-IV protocol, the international collaborative treatment protocol for children and adolescents with Langerhans cell histiocytosis.

**Conclusions:** Langerhans cell histiocytosis is a rare disease that may present with involvement of the frontal and ocular regions. Although periorbital cellulitis is initially treated with first-line medical therapy, if there is no improvement, radiological assessment and subsequent histological examination become essential for prompt diagnosis and appropriate therapeutic intervention.

## 1. Introduction

Langerhans cell histiocytosis (LCH) is a rare disorder with an incidence of 5–10 cases per million children per year and 1-2 cases per million adults per year, with a male-to-female ratio of 1.2:1 [1]. It is more common in the pediatric population, particularly among children aged 1–3 years, with a male predominance [2].

LCH is characterized by abnormal expansion of myeloid precursors that differentiate into CD1a+ and CD207+ cells in lesions [3]. The etiology and pathogenesis of LCH have been the subject of decades-long debate, but in the past 10 years, LCH has been re-evaluated as a myeloid neoplastic disorder arising from myeloid precursors [4]. This neoplastic perspective opens the door to the development of targeted therapies. The discovery of the somatic BRAFV600E mutation provides a molecular tag for lineage tracing to test the hypothesis that LCH could arise from hematopoietic precursors [1].

LCH presents with a wide range of clinical manifestations, from single indolent lesions to aggressive multisystem (MS) disease. The current classification system is based on the site of lesions, the number of involved sites (single system [SS] or MS/localized or multifocal), and whether the disease involves risk organs (hematopoietic system, liver, or spleen) [1].

The skeleton is the most commonly affected system, with bone lesions present in approximately 80% of patients with LCH, and in half of these cases, the lesions are solitary [1]. The most common site of bone involvement is the skull, followed by the spine, limbs, and pelvis [1, 5]. Patients with involvement of facial bones and bones of the anterior and middle cranial fossa (zygomatic, orbital, temporal, sphenoidal, and ethmoidal) are considered at risk for intracranial extension of the disease and central nervous system (CNS) involvement [6]. The differential diagnosis includes malignancies such as Ewing sarcoma, lymphoma, leukemia, and metastases, as well as osteomyelitis. A bone marrow biopsy or a biopsy of one of the lesions is often required for diagnosis [7].

Imaging (CT scan and MRI with contrast enhancement) plays a crucial role in the diagnosis and management of LCH, especially in the case of bone lesions. Given the unpredictable course of this disease, routine imaging must be performed long after resolution to rule out recurrence [8].

Current treatments include local therapy with surgery or chemotherapy, depending on the extent of the disease, particularly for lesions in proximity to the CNS, termed “CNS–risk” lesions [9].

## 2. Case Report

**Figure 1:**
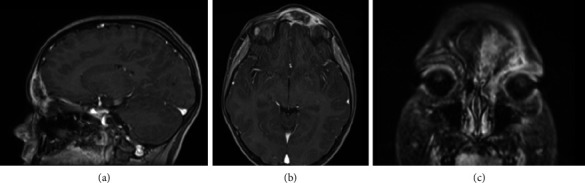
Pretreatment maxillofacial MRI ((a) sagittal view, (b) axial view, and (c) coronal view) showing a left frontoethmoidal lesion involving the orbital roof, measuring 25 × 20 mm, with inhomogeneous contrast enhancement, affecting the posterior wall of the frontal sinus and the orbit.

**Figure 2:**
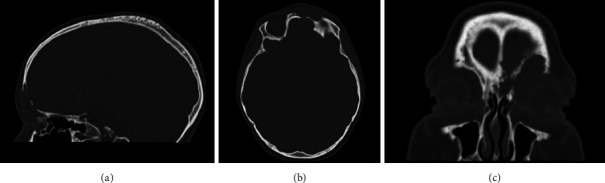
Pretreatment maxillofacial CT scan ((a) sagittal view, (b) axial view, and (c) coronal view) showing a resorption of the frontal bone wall.

**Figure 3:**
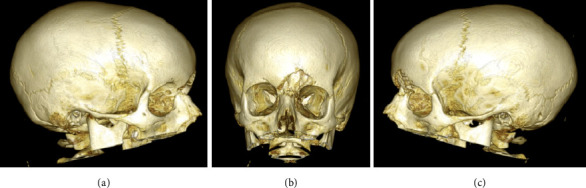
3D maxillofacial CT scan reconstruction allows for a better visualization of the extent and location of the frontal bone resorption.

We report the case of a 9-year-old boy presented to the emergency room with painful, left periorbital swelling. In the suspicion of periorbital cellulitis, he underwent medical therapy with prednisone 25 mg/die and ceftriaxone 1 g/die. Due to the lack of improvement, he returned to the emergency room 2 weeks later and came to the attention of the otolaryngologist. The endoscopic evaluation did not show pathological findings, but due to the age of the patient, the lack of response to the medical therapy, and the clinical presentation, along with normal laboratory findings, a maxillofacial MRI was then performed (Figures 1(a), 1(b), and 1(c)), showing a left frontoethmoidal lesion involving orbital roof, with inhomogeneous, contrast enhancement of 25 × 20 mm, involving the posterior wall of the frontal sinus and the orbit. Due to the unclear nature of the disease, a maxillofacial CT scan completed the radiological assessment, showing a resorption of all the walls of the frontal bone (Figures 2 and 3). Supporting Video 1 illustrates the three-dimensional reconstruction of the facial CT scan, highlighting the resorption at the level of the frontal bone wall.

The radiological findings indicated the necessity for a histological definition of the lesion. Consequently, the young patient underwent functional endoscopic sinus surgery (FESS) to access the left frontoethmoidal lesion, and a sample of the mass was taken to determine its nature. The surgery was confined to the left nasal fossa, involving an uncinectomy, anterior ethmoidectomy, medial antrostomy, and frontal sinusotomy. A complete resection of the mass was not possible due to its difficult localization, and an endonasal approach was preferred to avoid causing any esthetic damage. See Supporting Video 2 for the surgical procedure.

**Figure 4:**
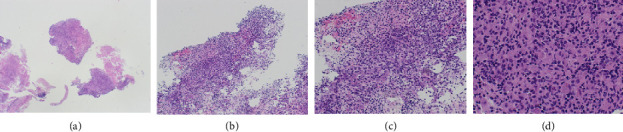
Histologic features of LCH. (a), (b) LCH lesion on hematoxylin and eosin staining x2; x10: foci of necrosis are seen. (c), (d) LCH lesion on hematoxylin and eosin staining x40: polygonal elements, large and elongate nuclei with prominent nuclear grooves, abundant pale eosinophilic cytoplasm, fine chromatin, and indistinct nucleoli admixed with eosinophils and neutrophils. Minimal nuclear atypia and low mitotic activity without atypical forms are shown.

**Figure 5:**
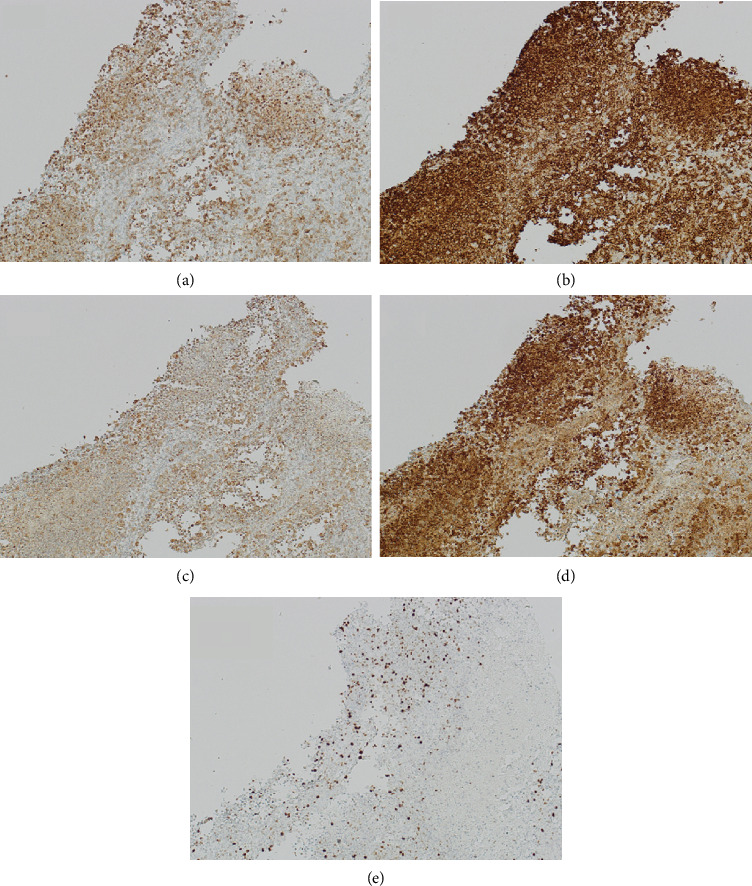
Immunophenotype of LCH cells (x10). (a) LCH cells show langerin-positive staining (granular cytoplasmic). (b) Typically, strong expression of CD1a. (c) In this case, LCH cells expressed the macrophage-associated marker CD68. (d) S100 protein-nuclear and cytoplasmic staining of the proliferating Langerhans cells is seen. (e) Proliferative index Ki67 (clone Mib1) is about 9%.

The histological examination was consistent with LCH. It revealed cells with large, elongated nuclei featuring prominent nuclear grooves, abundant pale eosinophilic cytoplasm, fine chromatin, and indistinct nucleoli, accompanied by eosinophilic and neutrophilic infiltrates. Minimal nuclear atypia and low mitotic activity without atypical forms were observed. Immunophenotyping was positive for langerin, S100, CD1a, and CD68 and negative for CD30. Molecular testing revealed negativity for BRAF, KRAS, NRAS, and PIK3CA (Figures 4 and 5).

The patient then underwent a total-body CT scan to assess the possibility of multifocal disease, and no other localizations were found. For staging purposes, an abdominal ultrasound was also performed, which revealed no pathological findings.

The patient was subsequently evaluated by a pediatric oncohematology specialist to enroll in the LCH-IV, the international collaborative treatment protocol for children and adolescents with LCH. The LCH-IV study aims to tailor treatment based on clinical presentation and response to therapy. Due to the disease's localization in the posterior wall of the frontal sinus and the orbital rim, and its proximity to the brain, the chosen treatment lasted 6 months, following the LCH-IV protocol. This included a baseline evaluation with a full blood count, blood chemistry, coagulation studies, and urine analysis.

The patient was initiated on prednisone 40 mg/m^2^/day orally, with reduction after 4 weeks and vinblastine 6 mg/m^2^ IV bolus weekly. After 8 weeks, a new MRI with contrast enhancement was performed to evaluate the response, which showed a positive clinical response. Hence, the treatment was then switched to maintenance with vinblastine every 3 weeks and prednisone for 5 days a week every 3 weeks. During this time, our patient suffered from one episode of mucositis, which delayed the administration of the dose at 1 month and one episode of myalgia at 3 months of treatment.

**Figure 6:**
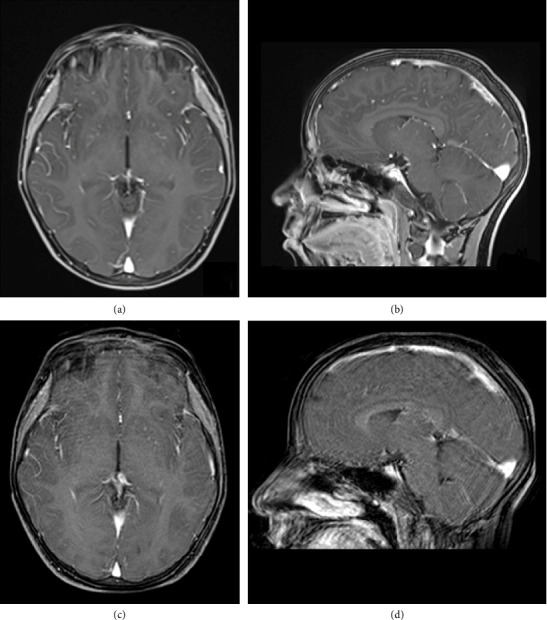
Comparison of MRI before treatment ((a) axial view and (b) sagittal view) and MRI 12 months after the start of treatment ((c) axial view and (d) sagittal view).

At the end of the treatment period, the MRI performed showed a complete regression of the lesion. The patient underwent hematological and radiological follow-up with no active disease at 12 months after the start of treatment (Figure 6).

## 3. Discussion

LCH is a rare hematological disorder resulting from an abnormal clonal proliferation of myeloid precursors. Currently, the classification of the disease comprehends SS disease (SS–LCH) and MS disease (MS–LCH), based on the extent of involvement. A monostotic lesion is the most common presentation of the disease with a predilection for the vertebral bodies, ribs, long bones, and calvaria, and 75% of these cases occur before the age of 20. It is rarely seen at the skull base. Clinical presentations are highly variable, with different symptoms based on sites of disease.

The frontal localization in literature counts only a few cases, described as isolated cases or counted among localizations in the cervicofacial region.

Publications from 2000 to 2023 were searched in PubMed. Keywords used in order to broaden the spectrum of the searched conditions were as follows: “Langerhans cell histiocytosis,” “pediatric,” “frontal bone,” “craniofacial bone,” “periorbital cellulitis,” “skull,” and “frontal sinus.”

**Figure 7:**
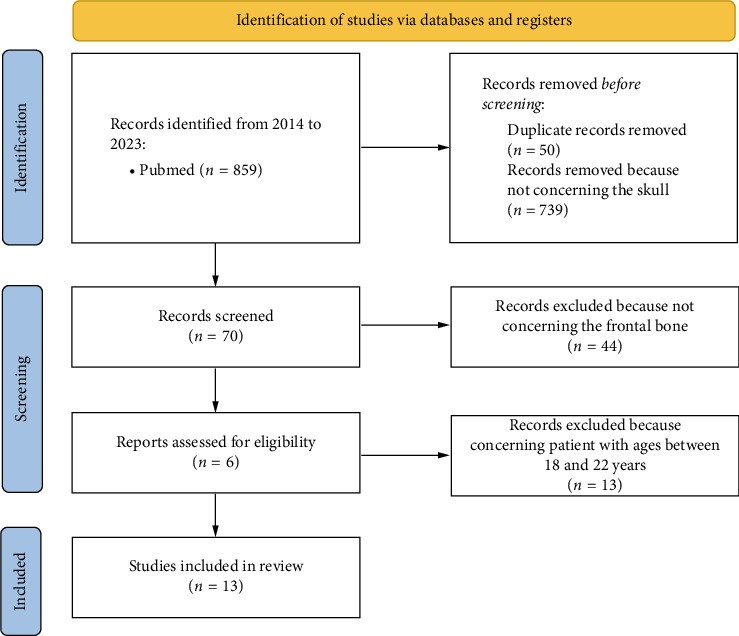
PRISMA 2020 flow diagram of literature review of pediatric frontal bone LCH.

During the database screening, 859 potentially relevant articles were found. After removing duplicates (50 articles) and articles not concerning the skull (739 articles), 70 articles were qualified for analysis. After excluding 54 articles because they were not concerning the frontal bone, 26 articles were accepted for full-text analysis. Finally, having taken into account the eligibility criteria and excluding articles about patients with ages between 18 and 22, all the 13 articles were accepted for review (Table 1).  Figure 7 shows the study screening process.

Most of the articles (38%) were published in 2015, 23% in 2016, and the remaining 39% were published in other analyzed periods, with the majority being published after 2016. Of the total articles, 4 were case reports on frontal bone LCH (31%), while 9 also reported cases involving other localizations (69%). Among the articles that reported patient age (all the “case reports” and five of the nine other articles), the average age was 6 years (assuming the case report of an “adolescent” referred to a 16-year-old patient). The remaining 4 articles reported only the findings of an LCH localization in the frontal sinus of a pediatric patient. Given that the reviewed articles do not specify symptoms, prognosis, or specific treatments for patients with frontal LCH localization, the authors decided not to include it in Table 1.

Presumptive diagnosis of LCH is usually made on radiologic and clinical findings. Diagnostic imaging plays a major role in the management of patients with LCH. In the skull CT scan, the lesions are typically represented by areas of osteolysis with sharp borders, giving a characteristic “punched out” appearance [22]. A biopsy is mandatory to get a definitive diagnosis. Among the diagnostic criteria are the presence of CD68/163+ histiocytes positive for CD1a+ and/or langerin (CD207) in immunohistochemical (IHC) staining, as well as the cellular morphology (large cells, varying from round to oval shapes, with a coffee bean-shaped nuclear groove, lacking the branching typical of inflammatory dendritic CD1a1+ cells) [23]. The attitude toward the choice of treatment depends strictly on the characteristics of the disease, mainly the extent at the diagnosis (SS–LCH vs. MS–LCH) and the involvement of risk organs (hematologic system, spleen, and liver). Patients with SS disease confined to a single site usually require only local therapy or observation, whereas patients with more extensive disease require systemic therapy [1].

The laboratory workup should comprehend full blood count, biochemistry (liver and kidney function and electrolytes), and erythrocyte sedimentation rate. To assess liver involvement, especially in young children, an abdominal ultrasound is recommended.

Our patient was included in the LCH-IV study protocol, an international, multicenter, prospective clinical study for pediatric LCH in patients younger than 18 years old.

Patients with CNS–risk lesions are collected in the Group 2 of the Stratum 1 (initial treatment) study group and undergo treatment with first-line therapy with prednisone and vinblastine.

Prognosis depends on the involvement of risk organs (bone marrow, liver, and spleen) and the response to initial systemic therapy. SS–LCH has an excellent prognosis, with a survival rate of nearly 100%. The 5-year recurrence rate is < 20%, and the recurrence typically involves the same organ system of the initial presentation but may involve a different localization. Considering the typically favorable prognosis and the propensity for spontaneous regression of the bone lesions (typically within 1–3 months), mutilating surgery should be avoided and systemic treatment is not needed in most patients, preferring a wait-and-see approach [24]. In our case, the patient fulfilled the criteria for the Stratum I of the clinical trial (MS–LCH patients [Group 1] and patients with SS–LCH with multifocal bone or “CNS–risk” lesions [Group 2]), therefore receiving systemic medical therapy.

State of the art suggests a follow-up every 3 months in the first year, and then every 6 months for the first 5 years and then continued yearly.

LCH being a rare condition, the rapid detection of the diagnosis with the help of a multidisciplinary approach leads to the start of the correct therapy: radiologist, pathologist, and oncohematologist are primarily involved with the collaboration of other specialists.

## 4. Conclusion

In this clinical case, the symptoms reported by the patient faded, making the differential diagnosis difficult at first. When faced with a challenging differential diagnosis, imaging is the first weapon we can dispose of, although definitive diagnosis is established by lesion biopsy and IHC analysis. The diagnosis of frontal sinus LCH should be considered in a pediatric patient with findings of predominant periorbital swelling involvement, ill-defined bony destruction without periosteal reaction, and a soft tissue lesion on CT and/or MRI confirmed with histopathologic examination. The systemic staging in order to differentiate unifocal and MS–LCH is important for prognosis and treatment. A multidisciplinary approach is the key to a prompt diagnosis. The presence of international protocols for this disease allows standardizing the treatment and the follow-up in such a rare disease.

As far as for our little patient, the postoperative period was uneventful. After chemotherapy, the patient had resumed his routine activities and started attending school.

## Figures and Tables

**Table 1:** Literature review of pediatric frontal bone LCH (publication time 2000–2023) that met our inclusion criteria.

*n*	First author, year	Year	Sex, age (or mean age)	Frontal/total case
1	Rivera [10]	2014	—	1/39
2	Maia [11]	2015	—	5/14 (orbit/frontal)
3	Bhat [12]	2015	—	2/6
4	Kim [13]	2015	M, 17 months	1/1 (case report)
5	Bezdjian [8]	2015	—	1/201 (frontal and parietal)
6	Preto-Zamperlini [14]	2015	M, 11 years old	1/2
7	Khoddami [15]	2016	1 M and 1 F, 4 years old	2/30
8	Tsutsumi [16]	2016	F, 3 years old	1/1 (case report)
9	Esmaili [17]	2016	1 M and 1 F, 4,5 years old	2/6
10	Zhang [18]	2017	4 M and 2 F, 6 years old	6/18
11	Kanazawa [19]	2021	1 F, adolescent	1/1 (case report)
12	Lisičić-Konaković [20]	2021	F, 3 years old	1/1 (case report)
13	Matsushita [21]	2022	6 M and 2 F, 4,5 years old	8/22

## Data Availability

The data that support the findings of this study are available from the corresponding author upon reasonable request.

## Supporting Information

Supporting Information 1 Video 1: The video shows the 3D reconstruction of the facial CT scan, highlighting the resorption at the level of the frontal bone wall.

Supporting Information 2 Video 2: The video shows the steps of the endonasal endoscopic surgical procedure. In the first few seconds, left-sided unilateral periorbital swelling is visible. A turbinate reduction was performed to allow easier access to the frontal sinus. Then, a medial maxillectomy was carried out, and finally, the frontal recess was opened, where the lesion could be partially seen and biopsied. Upon palpation of the frontal region through the skin, the remaining portion of the lesion becomes apparent.
